# Mini experiences X Major allergies

**DOI:** 10.31744/einstein_journal/2026CE2542

**Published:** 2026-09-02

**Authors:** Jullia Eduarda Feijó Belluco, Paulo Eduardo Silva Belluco, Fabíola da Silva Maciel Azevedo, Maurício Domingues-Ferreira, Rosana Zabulon Feijó Belluco

**Affiliations:** 1 Centro Universitário Unieuro Brasília DF Brazil Centro Universitário Unieuro, Brasília, DF, Brazil.; 2 Universidade do Distrito Federal Escola Superior de Ciências da Saúde Brasília DF Brazil Escola Superior de Ciências da Saúde, Universidade do Distrito Federal, Brasília, DF, Brazil.; 3 Força Aérea Brasileira Brasília DF Brazil Força Aérea Brasileira, Brasília, DF, Brazil.; 4 Universidade de São Paulo Hospital das Clínicas Faculdade de Medicina São Paulo SP Brazil Hospital das Clínicas, Faculdade de Medicina, Universidade de São Paulo, São Paulo, SP, Brazil.

Dear Editor,

A recently published article in the journal *Dermatitis* has caused us significant concern. The paper describes a series of toys marketed abroad under the name "Miniverse™ Make It Mini Sets".^(1)^ We believe it is necessary to alert pediatricians and other specialists, including allergists and dermatologists, about the risks associated with these kits intended for young children. The reality described in that letter equally applies to Brazil, as these toys, imported from China, are widely available in our market. In fact, a major national retailer advertises them as "the most desired toy in the world…".

These products contain liquid resin monomers. Children are instructed to pour the material into the provided molds and then cure it through exposure to ultraviolet light. This process encourages the creation of miniature food, utensils, and similar items. The product is indicated for children aged 8 years and older.^(1)^

These resins contain acrylates such as 2-hydroxyethyl methacrylate (HEMA), isononyl acrylate (IBOA), and 2-hydroxyethyl acrylate (HEA). However, acrylic compounds present in commercial products may be impure and contain undeclared acrylates.^(2)^ The "curing" process results in polymerization into plastic resins, which is considered inert. Nevertheless, monomeric forms of acrylic compounds released due to incomplete curing are well recognized for their sensitizing potential and capacity to induce allergic contact responses.^(3)^

Acrylates are widely used in a variety of applications. In healthcare, they are used in dental restorations, surgical adhesives, wound dressings, hearing aids, and other devices. In the cosmetic field, they are present in gel nails, artificial eyelashes, and hair extensions. ^(3)^ The surprising development is their presence in toys.

These acrylates have been increasingly recognized as important contact allergens. Their clinical relevance is evidenced by the inclusion of HEMA in the European Baseline Series in 2019. Currently, most cases of allergy to these substances are related to nail cosmetics, either in consumers or nail technicians.^(4)^ In studies in which patients were tested with a series of acrylates, HEMA was the most reactive substance.^(4)^ Most patients with allergy to acrylates/methacrylates demonstrate multiple sensitizations upon testing. This is due to cross-reactivity among structurally related acrylates and methacrylates. Sensitization to only one acrylate is uncommon, although this pattern is particularly described with IBOA.^(2)^

Reports of asthma related to methacrylates are not uncommon.^(2)^ Cases of airborne allergic contact dermatitis are frequently described.^(4)^ This fact may have implications for the younger siblings of children handling these products. Thus, children may develop sensitization without directly manipulating the toy.

Allergic contact dermatitis affects up to 20% of children. Its prevalence is underestimated because many cases are not diagnosed or not sufficiently valued to warrant appropriate diagnostic investigation.^(5)^ When untreated and refractory, it may lead to significant negative consequences for the children's quality of life. Therefore, prompt referral for patch testing is essential for adequate management.^(5)^ The issue is that the currently available pediatric patch test series in Brazil do not include HEMA, the main marker of acrylate allergy. Furthermore, IBOA, which is commonly found in glucose sensors and insulin pumps used in the management of type 1 diabetes, is not available even in adult patch test series. It is vital to reflect on the consequences for a child sensitized to IBOA through "play" who later develops diabetes.

A recent study by the European Society of Contact Dermatitis proposed a pediatric baseline patch test series.^(6)^ The authors used a 1% positivity rate as the threshold for inclusion in the baseline series. HEMA did not reach this threshold, showing positivity in 0.7% of girls (whereas no boys reacted to HEMA). However, the authors of that study emphasized that this is an allergen of special interest because of the progressively increasing use of artificial nails at earlier ages. This suggests that HEMA may be considered in the future for inclusion in adolescent female patch test series^(6)^ However, it is alarming to observe young children handling such substances.

In 2024, some of these products were recalled from the United States market because they contained acrylate levels exceeding permitted limits.^(1)^ The official communications cited cases of cutaneous irritation, including burns and exacerbations of asthma. However, other kits continue to be sold, and new manufacturers are entering the market with similar toys.^(1)^

It is worth noting that the toy's instruction leaflet includes safety warnings, such as the need for adult supervision, keeping animals away from the area, avoiding skin contact during preparation and before the gel has dried, using the provided tweezers, avoiding handling with injured skin, and using the product in a well-ventilated area, among others. However, one may question whether children will strictly adhere to such detailed instructions.

Most importantly, the company strongly recommends the use of gloves and eye protection when handling the resin, however these items are not included in the kit. Studies have indicated that acrylates can penetrate latex gloves. Therefore, specialists recommend the use of double-layer nitrile gloves or polyethylene gloves worn beneath nitrile gloves when handling such substances.^(2)^ These scientific recommendations are directed toward nail technicians who use these products. Should such precautions be a part of everyday childhood play?

We believe that relevant medical societies should be aware of these new trends in children's play. Specialists need to better understand how children play today to identify which specific substances should be used in potential patch tests. Families must be alerted to the risks associated with handling chemical products that are far from being harmless. Therefore, we must all be aware that exposing a child to unnecessary sensitization risks may have lasting consequences for their future health.

## Data Availability

The underlying content is contained within the manuscript.
